# Uppsala University Hospital 300 years—a survey of the surgical development

**DOI:** 10.1080/03009730802579737

**Published:** 2009-02-04

**Authors:** Henry Johansson

**Affiliations:** ^1^Department of Surgical Sciences, University HospitalUppsalaSweden

**Keywords:** Medical history, surgical development and specialization, Uppsala University Hospital

## Abstract

Professor Lars Roberg, the initiator of the Nosocomium Academicum (1708), our first university hospital in Sweden, claimed that ‘no-one who does not understand surgery is a completely trained doctor’. However, it was not until the end of 19th century that modern surgery was born.

The Academic Hospital was opened in 1867, and at the turn of that century Uppsala had a flourishing period under the influence of Karl Gustav Lennander, professor of surgery. In 1889 he performed the first appendectomy in Scandinavia.

At the end of the 19th century the surgical tree began to branch out. In Uppsala gynaecology and obstetrics was the first to be an independent speciality (1891). It was followed by ophthalmology (1894) with Allvar Gullstrand as professor and head of the department. Gullstrand received the Nobel Prize in medicine in 1911. A separate department for diseases of the ear, nose, and throat was founded in 1916 with the Nobel laureate Robert Bárány as head.

Thoracic surgery began in Uppsala in the 1940s with lung surgery and was separated from general surgery in 1958 with Viking Olov Björk as head of the department. Björk introduced open heart surgery in Uppsala.

In 1951 reconstructive plastic surgery was organized by Tord Skoog, who devoted special interest to operations for cleft lip and palate surgery.

Neurosurgery was established in 1962, and Uppsala has held a prominent position in the development of modern neurointensive care.

During the 1970s general surgery became subspecialized into gastrointestinal, endocrine, and vascular surgery. At the same time fracture surgery was transferred to the orthopaedists, and urological surgery became an independent speciality. Transplantation surgery was introduced in Uppsala in 1967, when Professor Lars Thorén performed the first kidney transplantation. Today Uppsala has a leading position in transplantation of pancreatic islets cells.

## Introduction

For many centuries surgery was regarded as pure craftsmanship, but during the 18th century the position of surgery in the medical sphere came to be increasingly recognized. In Uppsala the professor of medicine Lars Roberg, initiator of the Nosocomium Academicum (1708), claimed that ‘no-one who does not understand surgery is a completely trained doctor’. During the later part of the 19th century surgery was still looked upon as an ‘external activity’, and it was not until the end of that century that modern surgery was born, that is, just over 100 years ago. This was largely made possible by the fact that at that time the three great enemies of surgery, namely haemorrhage, pain, and infection, began to be overcome.

## Pioneer operations in Uppsala at the end of the 19th century

**Figure 1.**
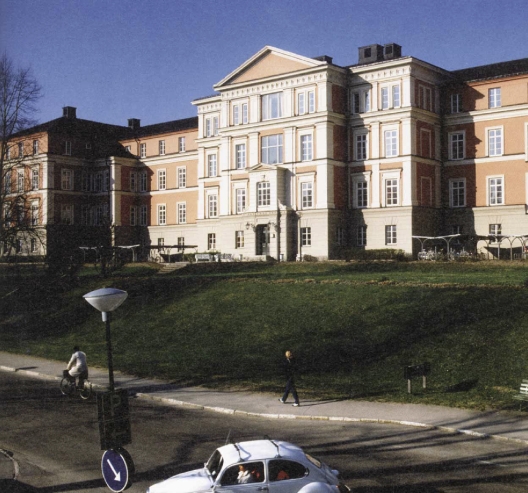
The Academic Hospital in Uppsala. The original building. Photo: Academic Hospital.

**Figure 2.**
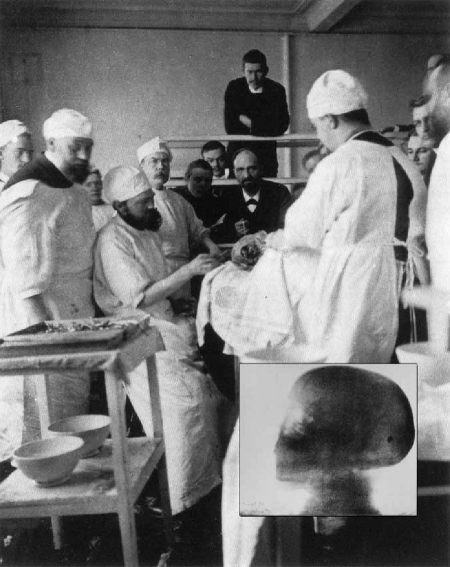
Professor Karl Gustav Lennander in 1897, removing a pistol bullet from the occipital lobe of the brain in a young man after the foreign body had been localized by X-ray. Photo: Medical Historical Museum, Uppsala.

Anaesthesia and asepsis came to create possibilities for the ‘first great development’ of surgery, which took place during the last decades of the 19th century. This applied particularly to abdominal surgery. The Academic Hospital (Figure 1) was opened in 1867, and at the turn of that century Uppsala had an especially flourishing period under the influence of Karl Gustav Lennander, professor of surgery. He performed the first appendectomy in Scandinavia in 1889, when he saved the life of a 28-year-old male student with a gangrenous appendicitis. Lennander was also the first in Sweden to undertake gastrectomy for cancer (1884), cholecystectomy (1889), and gastroenterostomy for peptic ulcer (1891). Lennander is also known for his ‘pioneer’ neurosurgical operation in 1897, when in a young man he removed a foreign body (a pistol bullet) from the occipital lobe of the brain after localizing it by X-ray examination (Figure 2).

With the development of surgery at the end of the 19th century, the surgical tree began to branch out. In Uppsala gynaecology and obstetrics was the first to break loose as an independent speciality, and this was given its own professorship in 1891, with A. O. Lindfors as the first holder. He was a skilful surgeon, and during his professorial period in Uppsala he performed no fewer than 17 caesarean sections, including one for eclampsia. At that time eclampsia was not considered to be a condition that should be treated by caesarean section, but Lindfors saw the possibility, and with his intervention three lives were saved—the mother and her twin offspring. The obstetric department later came to contribute with notable discoveries in reproduction research, and the years 1960–75 with Carl Gemzell as professor can well be said to have been days of glory for the department.

## Ophthalmology branched off early from the surgical tree

In Uppsala ophthalmiatrics became an independent ‘surgical speciality’ at an early date. It was given its own professorship in 1894, with Allvar Gullstrand as professor. A Nobel laureate in 1911, his scientific studies concerned the dioptrics of the eye; his innovative concepts of refraction of light in the eye were pioneering in ophthalmology, and Gullstrand must be regarded as one of the foremost scientists in our history of research. The eye clinic later came to devote considerable clinical and scientific activity to questions of the wide-spread diseases cataract and glaucoma, and the advances in this field were to a great extent a result of collaboration between preclinical and clinical researchers. The importance of Uppsala scientists for the development of cataract surgery must be especially pointed out here, particularly the significant contributions made by Torvard Laurent together with the Hungarian physician Endre Balazs to the introduction of hyaluronan in ophthalmic surgery. In co-operation with Pharmacia, hyaluronan came to be introduced under the name Healon®. This and other viscoelastic substances are now used worldwide in all cataract surgery. In the year 2005, at the University Hospital 1,500 cataract operations were performed, and in the whole of Sweden over 70,000. These operations are performed today under local anaesthesia and take about 30 minutes in uncomplicated cases. The visual function is improved in over 90% after surgery.

## Surgery for hearing improvement

A separate department for diseases of the ear, nose, and throat was founded in Uppsala in the beginning of the 20th century, to be exact in 1916. The head of the department was the Nobel laureate Robert Bárány. At first the department was located in what is nowadays called *Slottskällan*, formerly an establishment for hydrotherapy. Here there were 12 beds, an operating theatre, and a laboratory. It was not until 1925 that the department moved to the present University Hospital. The Department of Otolaryngology was a centre for otoneurological research even during Bárány's time, and later under the leadership of Hans Engström (1968–79) successful internal ear research was carried out there. Great interest was devoted to Menière's disease by Jan Stahle and his collaborators in the 1970s to 1980s.

At the end of the 1980s, so-called reconstructive middle ear surgery was developed in Uppsala, and the University Hospital became an important referral centre for a large part of the country. The aim of the surgical operations was to restore sound-transmitting parts of the external and middle ear that had been damaged by infections (chronic otitis) or congenital defects. New surgical principles and techniques were developed (tympanoplasty) with the aim of restoring the auditory ossicles and the tympanic membrane. During the same period surgery for acoustic neurinoma (e.g. vestibular schwannoma) was also developed. By surgical removal of the tumours via the ear, it was possible to avoid having to move aside the cerebellum, something that was hazardous and not seldom gave rise to serious complications; in particular there was a risk of damaging the facial nerve. This is the current method today, and the operation is always carried out in collaboration with neurosurgeons. Uppsala today is a national centre for this type of activity.

The development of brainstem and cochlear implantation has revolutionized the treatment of deafness. As early as in 1993 the first patient in northern Europe to receive a brainstem implant underwent this operation in Uppsala, and cochlear implantation surgery was developed in the present century. The difference from brainstem implantation is that in this form of surgery the electrode plate is inserted surgically into the cochlea instead of on the brainstem (nucleus cochlearis).

## Thoracic surgery is developed and the modern coronary vascular surgery is introduced

Thoracic surgery began in the 1940s with lung surgery, which was made possible by the introduction of the ventilator and intubation anaesthesia. It was mainly pulmonary tuberculosis that was treated. The first cardiosurgical operations consisted of closed commissurotomies in mitral stenosis, a disease which was usually a consequence of rheumatic fever. Simple congenital heart defects, especially defects of the atrial septum, could be operated on under moderate hypothermia without the help of a heart-lung machine. The first successful operation with the heart-lung machine was carried out in the USA in 1953, and in the following year the second one was successfully performed by Clarence Crafoord and collaborators at Sabbatsberg Hospital in Stockholm. The development of extracorporeal circulation proceeded rapidly after these successes, but the surgical treatment of coronary artery disease—bypass surgery—started at a later stage.

Thoracic surgery at the University Hospital was initiated at the beginning of the 1950s by Paul Rudström at the Department of Surgery. At that time it was mostly a matter of lung surgery. Gradually thoracic surgery came to be separated from general surgery, and in 1958 it was given an independent position with the dynamic Viking Olov Björk as head. Björk, who was one of Crafoord's pupils, continued with lung surgery, but most of all he came to develop open heart surgery in Uppsala. Together with Martin Holmdahl and the anaesthesiologists he also developed the method of deep hypothermia which was introduced after experimental trials in dogs. With deep hypothermia operations could be performed on the open heart, and in Uppsala this technique was used in a large number of patients.

During Björk's ‘Uppsala period’ heart valve surgery acquired a prominent position. Björk was one of the early surgeons to use ball valves for replacement of damaged valves, but he made his great contribution in this field when later, together with an American engineer, he came to construct the Shiley-Björk prosthesis, a prosthesis with a disc design. This became widely accepted, and during the end of the 1970s and the beginning of the 1980s it was the most frequently used prosthetic valve in the world.

After eight years in Uppsala Björk left our hospital to succeed Crafoord as professor in Stockholm. Even if Björk was only here for a relatively short time, that time must be regarded as a successful period in the history of the hospital.

Gradually patients with coronary sclerosis became predominant, and increased knowledge was gained about the relation of this sclerosis to cardiac infarction and angina pectoris. In Uppsala it was mainly Lennart Johansson, another Crafoord pupil and Björk's successor, who introduced the modern coronary vascular surgery at the hospital. At the end of the 1970s the need for this surgery increased explosively, and the University Hospital held the responsibility for the whole of northern Sweden, that is, from Uppsala to Treriksröset, with a catchment population of 2.5 million.

The original surgical treatment of arrhythmia consisted of pacemaker therapy. Today a large number of patients are treated with implanted defibrillators (ICD), and the use of biventricular pacemakers in certain forms of heart failure is another prominent example of the development. Surgical treatment of atrial fibrillation with the so-called Maze operation was carried out at an early date in Uppsala, which today has the largest number of patients in this field, with good results throughout. One development here is the use of pulmonary vein ablation, which can either complement other cardiac surgery or be conducted independently with a minimally invasive technique (so-called mini-Maze).

## Reconstructive plastic surgery

The demand for reconstructive surgery first became clearly evident during the First World War, in 1914–18. The war in the trenches on the western front, for example, resulted in a countless number of severe gunshot and blast injuries to the face in young soldiers, which required specialized treatment. This came to form the embryo of plastic surgery. The development of this new speciality continued during the interwar period, but it was not until the mid-1940s that the first department of plastic surgery was established in our country. This was at the Serafimer Hospital in Stockholm.

**Figure 3.**
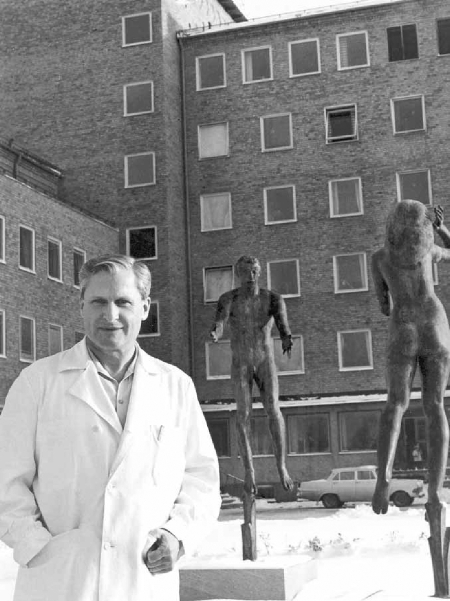
Tord Skoog, professor of plastic surgery in Uppsala 1951–77. Photo: Department of Surgical Sciences, Plastic Surgery, University Hospital, Uppsala.

In Uppsala plastic surgery became an independent speciality just a few years later. It was established on the initiative of the Uppsala surgeon Tord Skoog (Figure 3), who after training in Finland, England, and the USA organized a special department for plastic surgery in 1951. Skoog was a ‘surgical artist’, and his activity acquired international proportions. In 1960 Skoog also became the first professor in plastic surgery in Sweden. An area in which he came to devote special interest was the reconstructive surgery required for repair of cleft lip and palate in children.

### Cleft lip and palate surgery—an Uppsala model

The artistic element in the plastic surgical treatment of cleft lip and palate came to light when Skoog together with his collaborators described their own refined techniques for use in children with such malformations. These techniques became widely accepted both nationally and internationally. At the same time it became clear that the treatment of facial deformities requires well developed collaboration between several specialities. Such a collaborative treatment programme for children with these malformations was initiated in Uppsala in the mid-1950s and is still used by the team that takes care of children with cleft lip and palate and other facial deformities.

With the availability of refined methods for plastic surgery of the lip and nose in children with cleft lip and palate, the need for improved reconstructions of the jaw gradually became evident. The treatment of a cleft malformation involves a series of operations during the whole of childhood and adolescence. At first reconstruction of the cleft in the palate often resulted in incomplete closure, and a breakthrough in the treatment of this cleft was therefore achieved when it became possible to fill the defect with a graft of spongy bone with its surrounding periosteum. With this procedure complete coverage of the palate cleft was achieved. Today this is the absolutely predominant method for complete jaw reconstruction in facial malformations.

Skoog was also behind the initiative of the department of plastic surgery in allowing scope for the increasing care of burns. Previously burns injury patients had been treated within the frame of the department of surgery. The new department was the first in the country to organize holistic treatment of burns; that is, the patient was given intensive care concomitantly with rehabilitative measures. During the subsequent decades, under the leadership of Gösta Artursson, the majority of the principles–including operating at an early stage—that are still applied today in the treatment of patients with burns were established. Today Uppsala is one of our two burns centres—Linköping is the other.

## Uppsala acquires a department of neurosurgery

The development of neurosurgery in Sweden began in the early 1920s at the Serafimer Hospital in Stockholm, under the leadership of Herbert Olivecrona. In Uppsala a professorship in neurosurgery was established in 1962 with Einar Bohm, educated in Stockholm, in the Chair. The new department was located in ‘the old lung clinic’. Broad neurosurgical activity was built up and included the principal areas of tumour surgery (brain and spinal cord tumours), traumatology (treatment of injuries to the brain, spinal cord, and nerves), vascular surgery (treatment of arterial aneurysms and vascular malformations), and functional neurosurgery (pain surgery, surgery for epilepsy, and stereotaxy). In 1974 the department moved to the premises which are used today for neurosurgical activity.

Uppsala has held a prominent position in the development of modern neurointensive care. It was Urban Pontén who in the beginning of the 1980s introduced the use of intracranial pressure recording and long-term respirator treatment of unconscious patients to prevent the development of secondary brain damage—a therapeutic method that was developed in close collaboration with colleagues at the department of anaesthesiology.

Uppsala was given Sweden's first real department of neurointensive care (NIVA) in 1990. The modern intensive care came to play a major role in the great expansion of neurosurgery in the 1990s, which for Uppsala meant that neurosurgery came to account for a large proportion of the national and regional medical care undertaken by the University Hospital. An intracerebral microdialysis technique for chemical monitoring of some essential metabolites of the brain was introduced in 1992—the first in the world—which meant a breakthrough for the method and for neurointensive care, and gave Uppsala a leading position internationally. Standardized care and computerized surveillance have meant that the treatment results have progressively improved. Today about 600 patients receive care at NIVA every year. The most common conditions treated are head and spinal cord injuries (*circa* 35%) and haemorrhages in the nervous system (*circa* 35%).

## Further subspecialization of general surgery improves the care

During the 1970s in Uppsala, as in many other places in Sweden, the surgical activity became subspecialized, as a consequence of the accelerated development that had occurred in the medical sphere. Gastrointestinal surgery, endocrine surgery, and vascular surgery developed as separate areas within general surgery. It was no longer possible for surgeons to master ‘everything’. The surgeon became part of a team, and since that time the subspecialization has continued and will most certainly become even more pronounced in the future.

At approximately the same time as these changes took place, fracture surgery in Uppsala, which constituted a large part of the general surgeon's daily work, was transferred to the orthopaedist, and urological surgery became an independent speciality. The last fracture operation—nailing of a femoral fracture—in the surgical department was performed a few days after the department had moved its activities to the present ‘surgical block’, in 1970. The patient was included, so to speak, in the move.

### Gastrointestinal surgery—a speciality with an explosive development

The development in gastrointestinal surgery has been almost explosive in the last twenty years with the introduction of laparoscopic surgery. When this technique began to be used in the early 1990s—at first in cholecystectomy for gallstones—Uppsala was one of the hospitals that introduced the method for routine practice, and today the laparoscopic technique is applied in many areas in abdominal surgery.

The part of gastrointestinal surgery that first became dissociated as a separate identity in Uppsala was the treatment of inflammatory intestinal diseases (ulcerative colitis and Crohn's disease). Major pioneering work was carried out in this area by Urban Krause, who built up a ‘centre of excellence’ to take care of these severely ill patients. This development became an embryo for the division of gastrointestinal surgery that we see today, where colorectal diseases are treated by one team and diseases of the upper gastrointestinal tract, liver, and pancreas by another. This separation was carried out in the early years in Uppsala and is found today in virtually all hospitals in Sweden.

**Figure 4.**
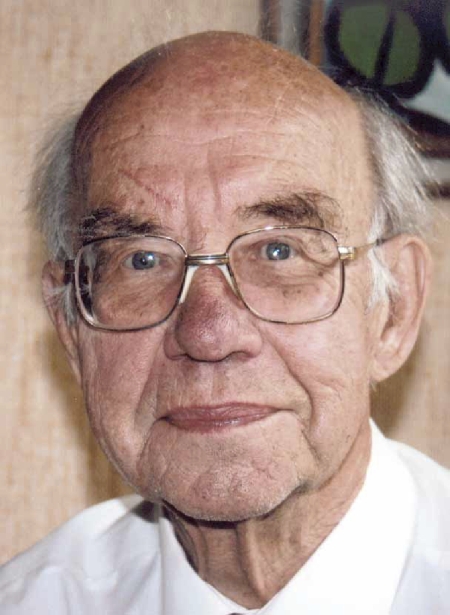
Lars Thorén, professor of surgery in Uppsala 1965–88. Photo: Åke Andrén-Sandberg.

It is now several decades since Uppsala began to show interest in surgery for obesity. In close collaboration with the geriatric department, nutritional studies were undertaken on patients with severe overweight who were offered surgical therapy. The pioneer in this field was Professor Lars Thorén (Figure 4), who at the beginning of the 1970s introduced intestinal shunt surgery—jejunoileal bypass—in Uppsala. Gradually this method for surgical treatment of overweight has been abandoned, mainly for reasons of late metabolic disturbances. As an alternative, different surgical techniques for decreasing the size of the stomach and achieving an early feeling of satiety have been developed, and the method most often used today is gastric bypass. In Uppsala important metabolic research has been carried out for this group of patients, and also the laparoscopic technique was adopted at an early stage for performing reconstructions of this type.

During the last twenty years of the 20th century the treatment of rectal cancer was revolutionized. The introduction of adjunctive treatment with irradiation and of a new, more exact surgical technique has led to an improvement of the survival of rectal cancer patients. Uppsala has held a leading position in this field and has initiated several large randomized studies that have changed the approach in rectal cancer therapy. The so-called ‘Swedish radiotherapy model’ that was introduced in the 1980s with 5 Gy for 5 days and surgery in the following week has been shown to be both an effective and cost-saving form of treatment. This radiotherapy model, which was initiated by the oncologist Sten Graffman in Uppsala, has now met with great interest all over the world, where it is referred to as ‘the Swedish model’. Uppsala was also the first centre in Sweden to apply the modern surgical technique (total mesorectal excision) in which an exact dissection in the anatomical plane is performed in rectal cancer surgery. By operating in this way and with selective use of irradiation, the risk of local recurrence is minimized, and it has been shown in Uppsala that the rate of local recurrence has decreased from 50% to less than 5% in the last twenty-year period.

At a pace with the refinement of the surgical technique, it has become important to gain increased knowledge of the sphincter function and of anal incontinence. In this area Uppsala has contributed to an understanding of how anal continence can be maintained by different surgical methods. This has led, among other things, to the introduction of a new sophisticated technique in which, for example, the gracilis muscle is transposed from the thigh and placed as a new closing muscle around the anus. The muscle function is then controlled with the aid of a pacemaker. As a further development in this area, a method of sacral nerve stimulation has been introduced, where the sacral nerves are stimulated with an electrode in a similar way. The Uppsala surgeons have contributed to the development of these surgical techniques.

### Endocrine surgery—surgery of tumours with varying biology and disease courses

In the development of endocrine surgery our own surgeons and clinical researchers have played a major role. Contributing to this have been the weight and breadth that endocrinology has attained in Uppsala and the strong position that endocrinological biochemistry and pathology have held over the years. Both of these subjects have had admirable representatives in Professors Leif Wide and Lars Grimelius. Wide has held a front-line position internationally in the development of various hormone analyses, and Grimelius's silver staining technique (published in 1968) is still the most useful for discriminating between an endocrine and a ‘non-endocrine’ tumour. It is used today at all histopathological laboratories working with endocrine pathology. Close co-operation with other disciplines has also been a prerequisite for the practice of endocrine surgery, and there has been well developed team- work between endocrinologists, radiologists, and oncologists. In Uppsala parathyroid surgery and surgery of endocrine abdominal tumours, in particular, have held prominent places, both nationally and internationally.

The parathyroid gland is somewhat of an ‘Uppsala’ organ. It was the Uppsala anatomist Ivar Sandström who discovered these glands in man in 1877, and he published his findings in *The Proceedings of Uppsala Läkarföreningen* in 1880. A hundred years later another unique study, by Göran Åkerström, was published from the department of surgery in Uppsala, on the anatomy and histology of the parathyroids. Hyperparathyroidism has been a comprehensive subject of clinical and experimental studies. The natural course of the disease has been mapped, and it has been shown that the increased mortality associated with hypercalcaemia and caused, in particular, by cardiovascular diseases can be reduced by successful surgery. In one interesting study it was found that no fewer than 65–80% of patients with hyperfunctioning parathyroid glands had psychiatric symptoms and that these problems could be eliminated by surgical treatment. In some recently published studies it has been demonstrated that primary hyperparathyroidism, even in a mild form, is associated with disturbances of both the lipid and skeletal metabolism, and that these are largely eliminated by surgery.

In the research on the pathogenesis of hyperparathyroidism, several important discoveries have been made here in Uppsala. It has been demonstrated, for example, that defective calcium regulation can be due to reduced expression of calcium receptors in the parathyroid cell. In addition, the research has shown that the disease can also be caused by a gene variant that lowers the expression of vitamin D receptors and thereby decreases the regulatory effect of the vitamin on the hormone secretion by the parathyroid glands, and growth. Another interesting observation is that the parathyroids possess an enzyme that can convert vitamin D to its active metabolite and hence regulate growth and hormone production. Benign tumours of the parathyroids—adenomas—show increased expression of this enzyme and thus inhibited growth, whereas cancers have reduced expression and thereby aggressive growth. This observation has pointed to the possibilities of treating malignant tumours in the parathyroid gland with vitamin D analogues.

Treatment of endocrine abdominal tumours is a national speciality of the Uppsala University Hospital. Moreover many patients come from abroad. Most patients are treated in collaboration between the oncoendocrinological clinic and the section of endocrine surgery. In Uppsala a surgical technique has been developed which makes it possible to operate on patients with large pancreatic tumours, even those with invasive growth into central abdominal vessels. In combination with effective cytostatic therapy, this has resulted in good survival and a good quality of life. Patients with multiple endocrine neoplasia (MEN), a hereditary syndrome with a dominant trait, often have several tumours concomitantly. Here it is important to determine whether the tumours are benign or malignant in order to plan the extent of the surgery. In genetic studies in Uppsala, mutations and chromosome losses that are typical of malignant tumours but are not observed in benign ones have been demonstrated. Studies in Uppsala have also shown that MEN patients should be operated on at an early stage to reduce the risk of tumour growth and metastasis.

Carcinoid of the small intestine, so-called mid-gut carcinoid, is a different form of tumour, which has received great interest in Uppsala, both clinically and in research. This form of tumour often gives rise to a difficult carcinoid syndrome with flush, diarrhoea, and connective tissue transformation of the heart valves. Lymph node and liver metastases are common. The regional lymph node metastases have a tendency to grow around the blood vessels supplying the small intestine, causing circulatory disturbances with severe abdominal anginal symptoms. In Uppsala this condition has been thoroughly investigated, and a surgical technique has been developed whereby the lymph node metastases can be removed and at the same time the vessels can be dissected free, allowing preservation of an intact circulation to the intestine. By this means the patients can also be relieved of disabling symptoms. For liver metastases so-called radio wave treatment has been developed, with which the metastases can be destroyed. The modern treatment in the form of both surgical and pharmacological therapy has meant that the survival among patients with malignant carcinoid has now increased considerably—from previously being approximately one year to almost ten years today.

### Vascular surgery—a relatively young speciality

Vascular surgery is a relatively young branch of the surgical tree, and for its expansion in the 20th century, especially in the later half, the developments in anaesthesiology, intensive care, diagnosis and pharmacology were prerequisites. The Uppsala professor in orthopaedics Tor Hierton had the foresight to recognize reconstructive vascular surgery as a way to reduce the risk of amputations, and right up to the beginning of the 1960s vascular surgery was part of the orthopaedic activity, after which time it moved to the department of surgery. In 1992 the first two professorships in vascular surgery were created in Sweden, in Uppsala and Malmö. The practical development has proceeded rapidly, and in Uppsala the physiological laboratory for vascular diagnosis is connected to the unit of vascular surgery, an arrangement which has advantages both for clinical practice and research, and has been a model for many other hospitals. Intimate collaboration with interventional radiologists for the purposes of endovascular treatment of vascular diseases has led to the creation in Uppsala of one of the first hybrid operating theatres in this country. To ensure new generations of vascular surgeons and provide a guarantee of their education, distance teaching has been initiated in Uppsala, with participants mainly from this health care region but also from other areas. This has become a model for other specialities. Regarding research there are several fields that are well represented, and research of international repute has been conducted on subjects such as aortic aneurysm, bowel ischaemia, vascular epidemiology, and prophylactic treatment of thromboembolism. In these areas the vascular surgeons in Uppsala are in the front line of research.

## Transplantation surgery is added

Transplantation surgery was introduced in Uppsala in 1967. It was in that year that Lars Thorén carried out the first kidney transplantation—three years after Professor Franksson in Stockholm had performed the first kidney transplantation in our country. For the last fifteen years there has also been a liver transplantation programme, which initially was conducted together with Huddinge Hospital but which Uppsala now runs under its own management.

The treatment of type 1 diabetes has made great progress in the last decades. In parallel with other forms of treatment of this disease, attempts have been made in the last forty years to transplant the pancreas. Such transplantations have been carried out in Sweden since 1974 and are an established form of treatment, which in combination with kidney transplantation has been found to improve the patients’ chances of survival. But it is a relatively large surgical procedure and not seldom leads to complications in the postoperative care. In recent years, therefore, fewer than ten combined transplantations of the kidney and pancreas per year have been carried out in Sweden.

### Transplantation of islets of Langerhans

Transplantation of islets of Langerhans, the endocrine parts of the pancreas, has always been considered an attractive possibility. The islets of Langerhans constitute only a small proportion of the pancreatic tissue but account for the entire production of insulin. Experimentally, techniques for islet cell transplantation were developed as early as in the 1960s, but it was not until the 1980s that the method gained clinical application. In our country, Uppsala has stood for the clinical development of this form of transplantation. Various techniques have been tried during the years, but for a long time the results were disappointing.

Unstable and refractory diabetes with frequent hypoglycaemias, often combined with absence of warning signals, has been the main target of islet cell transplantation. A standard has been developed in which the islets are introduced into the portal vein via a catheter. The islets then remain in the liver as small, fragile thrombi in the portal vein branches. The procedure can usually be carried out under local anaesthesia. After a few weeks the blood circulation to the islets is established through new formation of blood from hepatic vessels. During this time the patient receives intensive treatment with insulin—this to create optimal glucose control, a form of beta-cell rest, while the transplanted islets of Langerhans become adapted to their new milieu.

Since the autumn of 2000 Uppsala has been part of a Scandinavian network for clinical islet cell transplantation. The idea is that close collaboration within research and clinical application will lead to the attainment of a higher degree of efficacy after the transplantations. The patients’ insulin production often corresponds, despite repeated transplantations, to no more than 25–40% of the normal production. The reason for this is that the islets are subjected to a very rapid and deleterious thrombotic/inflammatory process, and as a result a large proportion of the transplanted islets are damaged and die. At present clinical studies are in progress to evaluate different strategies aimed at minimizing or completely blocking this reaction. The end goal stays firm: to attain a ‘normal’ insulin production as a regular result after a transplantation of islets of Langerhans.

## Fracture surgery—Uppsala surgery in focus

At the beginning of the 1970s Uppsala came to be somewhat of a national centre for modern fracture surgery. The man behind the modernization of fracture surgery in Sweden was Sven Olerud, then professor of orthopaedics in Uppsala. He had developed his interest in fracture surgery in the 1960s and subsequently took his activity to the department of orthopaedics when the treatment of fractures was transferred there. Among other methods Olerud introduced the AO technique, a Swiss method based on exact fracture reduction and stable internal fixation. Olerud and his collaborators became experts in ‘repairing’ difficult fractures, not least fractures of the pelvis and acetabulum. In several studies it was shown that this technique had clear advantages over the more conservative methods, which usually required a long period of plaster cast fixation. The modern technique, on the other hand, allowed immediate mobility training with quicker functional rehabilitation, which meant considerable advantages for the patient. There was no doubt that Uppsala came to be of great importance for the development of traumatology in our country. Contributing strongly to this was the driving force of the professor of surgery Lars Thorén in developing the modern fluid and nutritional therapy. Thorén's scientific contributions in the field of shock and fluid balance and his wide knowledge in these areas were also evident in his text-book on fluid balance, which became the source of information that was consulted in daily practice.

## Urology becomes an independent speciality

In Uppsala urological surgery became an independent speciality in 1974 with Åke Fritjofsson as head of the department. The main interest clinically and scientifically for the urologists has concerned questions about urinary bladder cancer and prostate cancer. The value of radical prostatectomy has been studied in a Scandinavian trial, where men with localized prostate cancer were randomized between radical prostatectomy and watchful waiting. It was the first study to show a survival benefit for men with prostate cancer treated with radical prostatectomy. In on-going studies of that cohort the quality of life outcomes of the choice of treatment strategy for men with localized prostate cancer are evaluated. In collaboration with the clinical chemist Gunnar Ronquist great attention has been paid to studying the role of prostasomes for the development of prostate cancer.

## General reflections

Surgery is and has always been dependent on different technological advances and on the development in other medical disciplines. For instance, improved knowledge in ultrasound and video techniques and the introduction of new instruments and implants have contributed to the development of surgery in the direction of ‘minimally invasive interventions’ in many fields. As a result the use of major open surgery has to a large extent decreased. Bypass operations have been largely replaced by balloon dilatations, and abdominal aortic aneurysms can be treated by application of prostheses with an endoscopic technique. Stenoses of different kinds can often be treated to advantage with stents that are introduced with radiological aid. At the same time surgery has become more organ-sparing and functionally adapted. Examples of this development are the breast-saving technique in breast cancer, sparing of the sphincter in rectal cancer, and preservation of the stomach in gastric ulcer. Nevertheless the radical ‘major surgery’ still exists and in some cases is necessary in the treatment of malignant tumour. Today it is carried out with safer methods and with better results than before. Moreover surgery is nowadays performed on increasingly older patients, and again in these older age groups very good results are achieved.

One of the great men of surgery, Theodor Billroth, is said to have stated at the end of the 19th century that the surgeon who goes in for operating on the heart has lost the respect of his colleagues for ever. Billroth could not have dreamed that a hundred years later we would not only be able to operate on the heart but would also be able to transplant it, as well as other organs, and do this with a high degree of safety and with good results. This shows how difficult it is to predict the future. It may be assumed, however, that the territorial boundaries in medical care will disappear. The care will become first and foremost focused on the patient, and the surgeon will increasingly become one person in a team; further, it is doubtful whether in the future we will have separate departments of surgery. So when in a hundred years the University Hospital celebrates its 400th anniversary, a chronicle of surgical activities will be quite different from that presented in this survey.

